# Cross-Linking Characteristics, Morphology, Dynamics, and Mechanical and Thermal Properties of Polychloroprene/Polybutadiene/Nano-Zinc (CR/BR/nZn) Compositions with Reduced Fire Hazard

**DOI:** 10.3390/ma16175804

**Published:** 2023-08-24

**Authors:** Aleksandra Smejda-Krzewicka, Przemysław Rybiński, Witold Żukowski, Dariusz Bradło, Kinga Wencel, Gabriela Berkowicz-Płatek

**Affiliations:** 1 Institute of Polymer and Dye Technology, Lodz University of Technology, Stefanowskiego Street 16, 90-537 Lodz, Poland; 2 Institute of Chemistry, The Jan Kochanowski University, Żeromskiego Street 5, 25-369 Kielce, Poland; 3 Department of General and Inorganic Chemistry, Cracow University of Technology, Warszawska Street 24, 31-155 Cracow, Poland; witold.zukowski@pk.edu.pl (W.Ż.); dariusz.bradlo@gmail.com (D.B.); gabriela.berkowicz@pk.edu.pl (G.B.-P.); 4 Department of Thermal Processes, Air Protection and Waste Utilization, Cracow University of Technology, Warszawska Street 24, 31-155 Cracow, Poland; kinga.wencel@pk.edu.pl

**Keywords:** nano-zinc, cross-linking, polychloroprene, polybutadiene, fire hazard, toxicity, Payne effect, hysteresis, STA analysis, sustainable development

## Abstract

The properties of unconventional blends of crystallizable and thermo-cross-linkable polychloroprene (CR) with polybutadiene (BR) were investigated in this study. The compositions were prepared using the method of reactive processing and cross-linking in the presence of nano-sized zinc (nZn). The purpose of the research was to assess the efficacy of nano-zinc as a curing agent of polychloroprene and polybutadiene (CR/BR) composites and to obtain rubber goods characterized by increased flame resistance. The blends were filled with nano-silica (aerosil) and fillers of natural origin (chalcedonite or silitin). The cross-linking process was characterized by determining the kinetics curves, the equilibrium swelling, and the Mooney–Rivlin elasticity constants. The morphology of the vulcanizate surface was specified by scanning electron microscopy (SEM). The dynamic and mechanical properties, flammability, and toxicity of gaseous substances involved in thermal decomposition were determined. Mass changes and thermal effects were studied using simultaneous thermal analysis (STA). It was confirmed that nano-zinc is an efficient curing agent for the polychloroprene and polybutadiene compositions, with a satisfactory degree of cross-linking (α_c_ = 0.10, CRI = 4.11 min^−1^), good mechanical strength (TS_b_ = 5 MPa), satisfactory tear resistance (T_s_ = 2.9 N/mm), and very high flame resistance (OI = 30%, HRR_max_ = 283 kW/m^2^). Filled products could be used as non-combustible materials, confirming the low fire hazard (1/t_flashover_ = 3.5–6.4 kW/m^2^∙s). The most effective filler of the tested composites was nano-sized silica.

## 1. Introduction

Polymers are among the most frequently used construction materials. This is due to their specific and interesting properties [1]. Polymers are commonly used in the production of machines and their components, furniture, buildings, trains, planes, buses, and cars. Many polymers are applied in the electronic and electrical industries as cables, transformers, bushings, channels, energy meter covers, supporting insulators, feeder pillar boxes, etc. [2]. Unfortunately, most polymers, both natural and synthetic, are combustible materials, and their use is associated with a high fire hazard. Fires occurring in industrial plants and in warehouses of finished polymeric products are among the most dangerous fires, and are characterized by a high burning rate. Fires involving polymeric materials and the release of toxic gases can destroy equipment, damage buildings, and cause losses of life or health.

The combustion begins with the ignition. When the appropriate amount of energy is supplied to the system, the temperature rises and self-accelerating oxidation reactions begin. A flame appears only when the fuel, oxygen, and energy necessary to initiate the combustion are present in the system at the same time. The course of the fire depends on the type and form of the materials undergoing the combustion, as well as on the conditions in which the fire starts. Fires pose a great threat to the lives and health of people and have a negative impact on the environment due to the generation of huge amounts of smoke, carbon black, and toxic products of thermal decomposition and combustion of polymeric products [3,4,5]. The composition of such toxic products depends on the amount and type of the burning material, as well as the oxygen amount and the ambient temperature. At low combustion temperatures, flue gases consist of pyrolysis products, while at higher combustion temperatures, secondary reaction products (such as: carbon monoxide—CO, carbon dioxide—CO_2_, nitrogen oxides—NO_x_, hydrogen cyanide—HCN, and hydrogen chloride—HCl) dominate in the gases [3,6,7]. Poisoning of the body by products of thermal decomposition of the burning material is the cause of up to 80% of fatal accidents occurring during fires. Such compounds easily penetrate the body through the respiratory tract and are absorbed through the skin [7,8]. The toxicity of combustion products is determined by the toxicity index (TI), which is defined as the numerical summation of the toxicity factors of selected gases produced by complete combustion of the material in the air. For each gas, the limit concentration (in ppm) at which it would cause the death of a person within 30 min is determined. For example, the concentration of carbon monoxide fatal to a person is 4000 ppm, and this value for carbon dioxide is 100,000 ppm [2].

The combustion involves many complex chemical and physical processes. During this process, simple and chain reactions occur at different rates. Most often, their products are fluorides, chlorides, and oxides. The burning of solids depends on their structures and proceeds through the hetero- or homo-phase mechanism. The hetero-phase process is based on the oxygen adsorption on the product surface during the combustion and flameless oxidation at the interface. The homo-phase combustion is caused by reactions of volatile decomposition products with oxygen in the absence of interfacial surfaces between the reactants [9].

The combustion of polymeric materials depends mainly on the morphology of their surface, the structure of the network, the cross-linking density, the concentration of substrates and products at each stage of combustion, the released heat, and the flame speed. In the case of elastomers, the degree of cross-linking has a large impact on the course of combustion. During the combustion of uncross-linked elastomers, significant amounts of liquid products are produced as additional sources of heat. The cross-bond formation between elastomers macromolecules during cross-linking and the generation of a three-dimensional network prevents this phenomenon [6,10]. The combustion of the elastomeric materials consists of their thermo-destruction (pyrolysis), ignition of the gas mixture, flame propagation, heat and radiation emission, smoke and toxic substances generation, and the possibility of self-extinguishing [5,10].

The parameter describing the fire of polymeric materials is their burning rate, which is measured using the linear burning rate (v_s_, m/s) and the mass burning rate (v_ms_, kg/m^2^∙s). The linear combustion velocity refers to the speed at which the flame front moves relative to the unburnt fuel. The mass burning rate determines the rate of polymer mass loss per unit area over time [6].

The flammability and high fire hazard due to limited thermal stability are major restrictions on the use of elastomers for many important applications. To improve the safety of cured rubber products, some selected substances must be applied to enhance the thermal, fire-retardant, and other performance properties of their final composites. In order to produce thermally stable materials, the grafting of polymers’ backbones with polymers exhibiting better thermal stability, the use of high-temperature polymer as a matrix, or the incorporation of a heat-resistant filler into the polymer are often used. In recent years, nano-sized fillers have been researched for the development of polymeric materials [11]. Studies have shown that nano-composites containing nano-fillers in very low concentrations (1–8 phr) have much better thermal stability than standard products. Layered silicates, graphene, and carbon nanotubes are popular research topics of many scientific studies [11,12,13,14]. Ho and co-workers have shown that the use of modified montmorillonite or carbon nanofibers as fillers in thermoplastic polyurethane elastomers (TPU) significantly reduces the flammability of the final products. The peak heat release rate of the neat TPU was 2290 kW/m^2^, and this parameter after using carbon nanofibers and nanoclay decreased to 624 and 664 kW/m^2^, respectively [15]. Zhou and co-workers studied thermoplastic polyurethane/DOPO derivative/sepiolite composites, which showed excellent fire performance compared to neat TPU. The blend containing TPU, DOPO derivative (DiDOPO), and sepiolite (87/10/3% mas.) achieved UL-94 V0 classes and a LOI of 34.4% [16]. The effect of the lignocellulose filler obtained from alkalized wheat straw and treated with di-potassium phosphate on the properties of natural rubber (NR) composites was described in the paper referenced in [17]. A lower flammability of NR vulcanizates filled in this way (max. heat release rate was 376 kW/m^2^) was found compared to unfilled NR (max. heat release rate was 511 kW/m^2^). However, a radical decrease in the flammability was obtained after using additive flame-retardant compounds (e.g., ammonium polyphosphate and aluminum hydroxide). In this case, the maximum heat release rate was 194 kW/m^2^.

In this paper, elastomeric blends consisting of polychloroprene and polybutadiene (CR/BR) are presented. The preparation of elastomeric compositions is often practiced in the elastomer industry, because it enables the manufacturing of new materials with unique properties. In addition, through the appropriate selection of rubbers, it is possible to obtain better processability of rubber blends, as well as to reduce the production costs of elastomeric goods. The unique properties of polychloroprene mean that it is often mixed with such rubbers as natural rubber [18,19]; carboxylated acrylonitrile-butadiene rubber [20]; styrene–butadiene rubber [21,22,23]; and, recently, with butadiene rubber as well [24,25,26,27,28,29,30,31,32]. Vulcanizates made of polychloroprene are characterized by their good tensile strength, resistance to weather conditions, thermal resistance up to 100 °C, and good resistance to ozone and non-polar solvents. The presence of halogen elements (-Cl) in the CR macromolecules makes them resistant to fire. Nevertheless, the disadvantage of polychloroprene is that it is quite difficult to process due to its susceptibility to crystallization [33,34]. Therefore, it is necessary to mix it with other elastomers.

In this work, polybutadiene (BR) was incorporated into polychloroprene (CR) in order to use nano-sized zinc in a controlled way to produce interelastomer reactions between the applied rubbers, and, consequently, to obtain rubber materials with very good properties. The cross-linking process of elastomeric blends containing polychloroprene and polybutadiene in the presence of nano-zinc is an unconventional method which has not been described in the literature so far. In studying scientific journals, no information was found on the use of nano-zinc as a curing agent for other elastomers. Therefore, the unknown cross-linking method of the CR/BR composites with nano-zinc that is described in this paper is an important element of scientific novelty. Due to the increased affinity of silica to polychloroprene, the nano-silica (aerosil) and silica of natural origin (Neuburg silica earth or chalcedonite) were used as fillers. Fumed silica was used as a filler of the CR/BR blends due to its high activity and very high specific surface area. It was expected that these silica properties and the presence of functional groups on its surface would enable the generation of filler–elastomer matrix interactions (especially silica–CR). The differences between highly active silica and biofillers (chalcedonite and sillitin) are a very interesting aspect of this paper, which is a continuation of our earlier published research [32]. In conclusion, the aim of this work was to produce new compositions of polychloroprene with polybutadiene (CR/BR), which is unconventionally cross-linked in the presence of nano-zinc (nZn) and characterized by increased flame resistance. The most important aspect of these studies is to determine the flame resistance of the CR/BR/nZn products and to assess the toxicity of gaseous substances formed during their thermal decomposition. So far, the toxicity of gaseous decomposition products formed during the combustion of CR/BR vulcanizates has also not been described in the literature. Therefore, the results presented in this work are of great practical importance. The production of a material with limited susceptibility to burning is desirable not only for safety reasons for humans, but also for ecological reasons; therefore, the purpose of our research fits well with the Sustainable Development Goals (2030 Agenda for Sustainable Development) [35].

## 2. Materials and Methods

### 2.1. Materials

In this work, two types of elastomers were used: polychloroprene, CR (type: BAYPREN^®^216) with 40% bonded chlorine content and Mooney viscosity of ML 1 + 4 (100 °C): 49 ± 5 (delivered by LANXESS GmbH, Köln, Germany), and polybutadiene, BR (type: SYNTECA^®^44) with 97% cis-1.4 units and Mooney viscosity of ML 1 + 4 (100 °C): 39 (delivered by Synthos S.A., Oswiecim, Poland). Nano-zinc (nZn), with a density of 7.13 g/cm^3^, an average particle size of 40–60 nm, and a surface area (BET) of 12 m^2^/g (delivered by Sigma-Aldrich Chemie, Steinheim am Albuch, Germany), was used as a cross-linking agent. The following fillers were used: nano-silica, Aer (fumed type: Aerosil^®^380), with an average particle size of 70 nm and pH = 3.7–4.5 (delivered by Evonik Industries AG, Essen, Germany); chalcedonite, Chal (type: M12), with an average particle size of 9 μm and pH = 6–8 (delivered by Crusil Co., Inowlodz, Poland); and sillitin, Sil (Neuburg silica earth, type: Z86), with an average particle size of 12.7 μm and pH = 8.5 (delivered by Hoffmann Mineral GmbH, Neuburg, Germany). Stearic acid, SA, with density of 0.94 g/cm^3^ (delivered by POCH S.A., Gliwice, Poland) was used as a dispersing agent.

### 2.2. Compounding and Vulcanization

The recipe for the compounding of the CR/BR blends is given in Table 1. These blends were created on a two-roll mill (type: Laborwalzwerk 200 × 450, Krupp-Gruson, Magdeburg-Buckau, Germany).

First, the plasticization of polychloroprene and polybutadiene was carried out. The components were added as follows: CR and BR, stearic acid, nano-zinc, and a filler. The mixing time for composites without a filler was 6 min, and for filled blends, 10 min, lasting until all ingredients were well mixed.

The produced blends were vulcanized in hydraulic presses in appropriate metal molds. The vulcanization parameters were a temperature of 160 °C, a pressure of 150–180 bar, and a duration of 30 min.

### 2.3. Characteristics of the Cross-Linking Process

The vulcanization process is characterized by determining the cure kinetics, the equilibrium swelling, and the Mooney–Rivlin elasticity constants.

The cure kinetics of the CR/BR/nZn blends were determined using the Alpha Technologies (MDR 2000) oscillating disk rheometer (Alpha Technologies, Hudson, OH, USA) at 160 °C (ASTM D5289-17 standard [36]), which was employed in order to determine the following parameters: scorch time (*t*_02_); vulcanization time (*t*_90_); minimal torque (T_min_); torque increment after 30 of the heating (∆T_30_), which is the difference between the torque after heating and minimal torque values; and cure rate index (*CRI*), designated according to Formula (1):

(1) $$CRI=\frac{100}{t_{90}-t_{02}}$$

Swelling behavior was assessed using toluene (according to ASTM D 471 [37]). From each vulcanizate, four test pieces of 25–60 mg of different shapes were cut out, weighed using an electrical balance, and swollen in toluene until equilibrium was reached (for 72 h). After this time, the swollen samples were removed from toluene and washed with diethyl ether, and their weights were determined again. The samples were dried to a constant weight at temperature of 50 °C and then reweighed.

Equilibrium volume swelling (*Q_v_*) was calculated using Formula (2):

(2) $$\;Q_{v}=Q_{w}\times \frac{d_{v}}{d_{s}}$$

where *Q_w_* is the value of the equilibrium mass swelling (mg/mg), *d_v_* is the vulcanizate density (g/mL), and *d_s_* is the solvent density (g/mL).

The degree of cross-linking (α_c_) was determined using Formula (3):

(3) $$\alpha_{c}=\frac{1}{Q_{v}}$$

The Mooney–Rivlin elasticity constants (2C_1_, 2C_2_) were calculated using the Mooney-Rivlin Equation (4):

(4) $$2C_{1}+\lambda^{-1}\times 2C_{2}=\frac{P}{A_{0}\times \left(\lambda -\lambda^{-2}\right)}$$

where P is the deformation force at λ (kG), λ is the deformation (λ = l/l_0_), l is the measuring section of the sample loaded with P (cm), l_0_ is the measuring section of the unloaded sample (cm), A_0_ is the cross-sectional area of the unloaded sample (cm^2^), 2C_1_ is the first elasticity constant (kG/cm^2^), and 2C_2_ is the second elasticity constant (kG/cm^2^).

### 2.4. Determination of Surface Morphology

The morphology of the vulcanizates was assessed using a scanning electron microscope (SEM). This was a Hitachi Tabletop Microscope TM-1000 (Tokyo, Japan) product. The preparation of the samples for measurement consisted of placing a double-sided self-adhesive foil onto a special table and gluing the testing sample to it. Then, a gold layer was applied to the prepared sample using the Cressington Sputter coater 108 auto vacuum sputtering machine (Redding, CA, USA) at a pressure greater than 40 mbar for 60 s. The samples prepared in this way were placed into the scanning electron microscope chamber, and the measurement was performed. The distribution of elements in selected micro-areas was performed using scanning electron microscopy by the Hitachi S-4700 (Tokyo, Japan), with the ThermoScientific (Waltham, MA, USA) energy-dispersive spectrometer (EDS) microanalysis adapter.

### 2.5. Determination of Dynamic and Mechanical Properties

For the vulcanizates, the following properties were determined: strength properties, hysteresis losses and Mullins effect, tear resistance, hardness, elasticity and loss modules, Payne effect, glass transition temperature, and mechanical loss coefficient.

Measurements of the tensile properties were carried out using a testing machine (Zwick1435/Roell GmbH & Co. KG, Ulm, Germany) [38]. The parameters determined from this test were: stress at elongation of 100, 200, and 300% (S_e100_, S_e200_, S_e300_); tensile strength (TS_b_); and relative elongation at break (E_b_). Each property was determined for five samples. The test was conducted at a constant speed of 500 mm/min.

The hysteresis losses were determined using a testing machine (Zwick1435/Roell GmbH & Co. KG, Ulm, Germany). Each test was conducted for three samples, which were stretched five times to 200% elongation at a stretching speed of 500 mm/min, and the initial force was 0.1 N. The Mullins effect was determined according to Formula (5):

(5) $$E_{M}=\frac{W_{1}-W_{5}}{W_{1}}\times 100\%$$

where *W*_1_ is the hysteresis loss at the first extension of the sample (N∙mm) and *W*_5_ is the hysteresis loss at the fifth extension of the sample (N∙mm).

The tear strength (T_s_) was tested by method A in accordance with ISO 34-1:2015 [39] using a testing machine (Zwick1435/Roell GmbH & Co. KG, Ulm, Germany). Rectangular specimens with dimensions of 100 mm × 15 mm and a cut of 40 mm were used for the tests.

Hardness (HA) was tested on the Shore A scale using a Zwick/Roell hardness tester according to ISO-48-4:2018. Each test was performed ten times. The samples were in the shapes of cylinders, with diameters of 80 mm and heights of 6 mm.

The dynamic properties of the vulcanizates were determined by the minimum and maximum storage moduli (G’_min_, G’_max_), the maximum loss modulus (G’_max_), and the Payne effect (ΔG’, from Formula 6) at room temperature. The test was performed using the Ares G2 rotational rheometer (New Castle, UK) according to ISO 4664:2011. The tested samples, in the form of discs with dimensions of 25 mm × 2 mm, were placed between special measuring plates of the apparatus. The parameters which were used were as follows: a soak time of 10 s, an angular frequency of 10 rad/s, a logarithmic sweep with strain from 0.005 to 70% s, 20 points per decade, and an initial force of 5 N.

(6) $$\Delta G^{\prime }={G^{\prime }}_{\max}-{G^{\prime }}_{\min}\;\;$$

where *G’*_max_ is the maximum storage modulus (MPa) and *G’*_min_ is the minimum storage modulus (MPa).

Dynamic mechanical analysis (DMA) was performed using a DMA/SDTA861e analyzer (Mettler Toledo, Greifensee, Switzerland). The analysis of the CR/BR/nZn vulcanizates was carried out in tension mode, in the temperature range from −150 °C to 70 °C, with a heating rate of 3 K/min, a frequency of 1 Hz, and a strain amplitude of 4 μm. The glass transition temperature (T_g_) was indicated by the maximum of the tan δ = f(Temperature) curve, where tan δ is the mechanical loss factor.

### 2.6. Simultaneous Thermal Analysis (STA)

The thermal analyses were carried out with use of the TG (thermogravimetry) and DSC (differential scanning calorimetry) thermal methods on STA 449 F3 Jupiter (NETZSCH, Waldkraiburg, Germany, Selb) equipment. A round, flat piece weighing about 15 mg was cut out of each sample and placed into the alumina (Al_2_O_3_) crucible. The measurements were taken according to an experimental procedure consisting of pyrolysis (1st, 2nd step) and combustion (3rd step): (1) isothermal stage at a constant temperature of 50 °C (2 min under inert gas N_2_ with gas flow rate of 100 mL/min); (2) heating from 50 to 1000 °C (20 °C/min, under inert gas N_2_ with a gas flow rate of 100 mL/min); and (3) isothermal stage at a constant temperature of 1000 °C (10 min under an oxidizing atmosphere of synthetic air, with a gas flow rate of 126 mL/min). The mass change and thermal effects occurring during the 2nd stage of the procedure are shown in the figures further in this work.

### 2.7. Determination of Fire Hazard, Flammability, and Toxicity

Flammability was designated by the oxygen index method using an apparatus built at the Institute of Polymer and Dye Technology of the Lodz University of Technology [40]. Samples with dimensions of 50 mm × 10 mm × 4 mm were placed into a quartz column. Using rotameters, the flow rates of the gases flowing through the sample were determined. The nitrogen flow rate was 400 L/h, while the oxygen flow rate was selected so as to determine its lowest concentration in the mixture of oxygen and nitrogen, where the sample are burned completely within 180 ± 10 s. The sample was ignited with a gas burner powered by a propane–butane mixture for 5 s, after which the source of fire was removed and the burning time was measured. The test was performed according to PN-ISO 4589-2. The oxygen index (*OI*) was calculated from Formula (7) [23,24,25,26,28,29,30,31,32]:

(7) $$OI=\frac{O_{2}}{O_{2}+N_{2}}\times 100\%$$

A cone calorimeter built by Fire Testing Technology was used to measure the ignition characteristics, heat release rate, and sample mass loss rate according to ISO 5660-1. An external radiant heat flux of 35 kW/m^2^ was applied. All the samples were measured in the horizontal position and wrapped with thin aluminum foil, except for the irradiated sample surface. The standard uncertainty of the measured heat release rate was ±10%. Based on the results, the fire hazard was calculated as the fire propagation rate (1/*t_flashover_*) from Formula (8) [30,32]:

(8) $$\frac{1}{t_{flashover}}=\frac{HRR_{MAX}}{TTI}$$

where *HRR_max_* is the maximum heat release rate and *TTI* is the time to ignition.

The toxicity of gaseous products of thermal decomposition of the CR/BR/nZn vulcanizates was determined using a laboratory set. The method used to determine toxicity (toxicometric indices) is described in detail in the previous articles of this study’s authors [32,41,42].

The results are presented as toxicometric indicators (*W_LC_*_50,_ *W_LC_*_50*M*_, *W_LC_*_50*SM*_) in specific emission units (*E*, g/g). The *W_LC_*_50_ index was the maximum toxic concentration of gaseous products forming during the thermal decomposition and combustion of the testing material at the temperature (T), according to Formula (9) [41]:

(9) $$W_{LC50}=\frac{LC_{50}^{30}}{E}$$

where *E* is the emission (g/g) and *LC*_50_^30^ is the lethal concentration of gaseous products of thermal decomposition and combustion of the testing material, which caused a 50% lethality of the testing animals during 30 min of exposure (g/m^3^).

The *W_LC_*_50*M*_ index is defined as the resultant of the *W_LC_*_50_ index of individual products of thermal decomposition and combustion for a given temperature, according to Formula (10):

(10) $$\frac{1}{W_{LC50M(T)}}=\sum_{}^{n}\frac{1}{W_{LC50}}$$

where *n* is the number of gaseous products.

The *W_LC_*_50*SM*_ index is defined as the arithmetic average of *W_LC_*_50*M*_ indices, according to Formula (11):

(11) $$W_{LC50SM}=\frac{W_{LC50M450}+W_{LC50M550}+W_{LC50M750}}{3}$$

## 3. Research Results and Discussion

### 3.1. Vulcanization Results of Unfilled and Filled CR/BR/nZn Blends

The vulcanization process is the chemical, permanent connection of the elastomer macromolecules by the cross-bonds leading to the network as a consequence of chemical reactions and physical processes. The type and amount of curing agents used determine the functional properties of the cured product [43]. The presence of specific functional groups with unique chemical activity is necessary for the vulcanization of the elastomers. The chemical structure of the rubber macromolecules determines the ability of the respective cross-linking agent to react with the rubber at a controlled rate. In these studies, an unconventional curing agent, nano-zinc, was applied. So far, nano-sized zinc has not been used as a curing substance, but these results show that it is possible. Since the vast majority of rubber materials are filled products, aerosil (Aer), chalcedonite (Chal), or sillitin (Sil) in the amount of 30 phr were used as fillers in the CR/BR/nZn blends (80/20/2.5 by wt.). Aerosil was selected as a filler with high activity and a very high surface area, and chalcedonite and sillitin were used as biofillers. Their use can lead to the production of biocomposites, which have important ecological aspects. The cure characteristics of the unfilled and filled blends in the presence of nano-zinc were investigated, and the results are shown in Table 2.

The cross-linking kinetics of the CR/BR/nZn compounds was tested by the vulcametric method, which demonstrated that the heating of these blends led to an increase in the “marching” torque (with moderate speed). The research shows that nano-sized zinc can be used as an effective curing agent; that cross-linked blends were characterized by a short scorch time (t_02_ = 1.98 min); and that, after the filling of the compounds, this parameter clearly increased (from 2.42 min for aerosil to 3.29 min for chalcedonite, and up to 5.38 min for sillitin). All tested blends, both unfilled and filled, were characterized by very similar vulcanization times of approx. 28 min, which shows that the fillers used herein do not have a significant effect on the vulcanization time. However, their presence distinctly changed the minimal and maximum vulcametric torque. The T_min_ value of the unfilled CR/BR/nZn composite was 0.56 dN·m. The use of chalcedonite or sillitin as a filler led to an increase in this parameter to values of 0.58 dN·m and 0.83 dN·m, respectively. The highest minimal torque (T_min_ = 4.09 dN·m) was observed for the aerosil-filled blend. The markedly higher viscosity in the case of this blend is most likely related to the fact that aerosil is a nanofiller with high activity and a very developed specific surface area. A similar relationship can be observed for the increase in torque increment after 30 min of heating (ΔT_30_). The highest value of this parameter (7.95 dN·m) was also observed for the CR/BR/nZn/Aer composite (Table 2). The probable cause of this phenomenon was the presence of a large amount of polar silanol groups on the aerosil surface, which can interact with the polar polychloroprene present in the tested blends. The chemical reaction between the allylic chlorine atoms of CR and the silanol groups of aerosil led to additional bonds in the elastomer phase [44,45,46]. Therefore, the aerosil-filled CR/BR/nZn blends were shown to possess insignificantly shorter vulcanization times compared to the blends filled with chalcedonite or sillitin.

The results obtained from the vulcametric measurements were confirmed by the swelling degree in toluene. The swelling data show that the unfilled CR/BR/nZn vulcanizate had a higher equilibrium swelling degree (Q_v_ = 9.70 mL/mL), which verifies the small degree of cross-linking in this case (α_c_ = 0.10). The presence of filler in the blends led to a decrease in the equilibrium swelling value. The Q_v_ value for the CR/BR/nZn/Aer vulcanizate was 6.21 mL/mL, which was a consequence of the highest degree of cross-linking (α_c_ = 0.16). The difference in the Q_v_ values for the tested vulcanizates resulted from their unequal degrees of cross-linking, which has previously been confirmed by the results obtained from vulcametric studies. The highest degree of cross-linking for the vulcanizate filled with aerosil was also confirmed in the Mooney–Rivlin study. The first elasticity constant (2C_1_), which was proportional to the network density, was 3.22 kG/cm^2^ for this sample. For vulcanizates containing chalcedonite or sillitin, the values of 2C_1_ were 2.28 kG/cm^2^ and 1.87 kG/cm^2^, respectively. The smallest value of 2C_1_ was found for the unfilled sample. The second elasticity constant (2C_2_)—as a measure of deviations from the ideal network—was in the range of 0.84–1.56 kG/cm^2^ for all vulcanizates (Table 2).

The results regarding the degree of cross-linking, determined from the vulcametric curves, the equilibrium swelling, and the Mooney–Rivlin equation, correlate well, and indicating that CR/BR blends can be effectively cured with nZn. For comparison, the degree of cross-linking of blends containing 80 phr of CR and 20 phr of BR vulcanized in the presence of nano-zinc oxide (nZnO) was lower (ΔT = 3.52 d∙Nm), and the curing time was longer (t_90_ = 40.12 min) [26]. Moreover, the use of aerosil, chalcedonite, or sillitin as fillers of the CR/BR/nZn compositions led to a greater degree of their cross-linking, with aerosil having the greatest positive effect. Chalcedonite and sillitin should be classified as passive fillers, so it is most likely that interactions with polychloroprene or polybutadiene are limited in their cases. Weak interactions are not conducive to increasing the degree of cross-linking.

### 3.2. Morphological Surface of Unfilled and Filled CR/BR/nZn Vulcanizates

**Figure 1:**
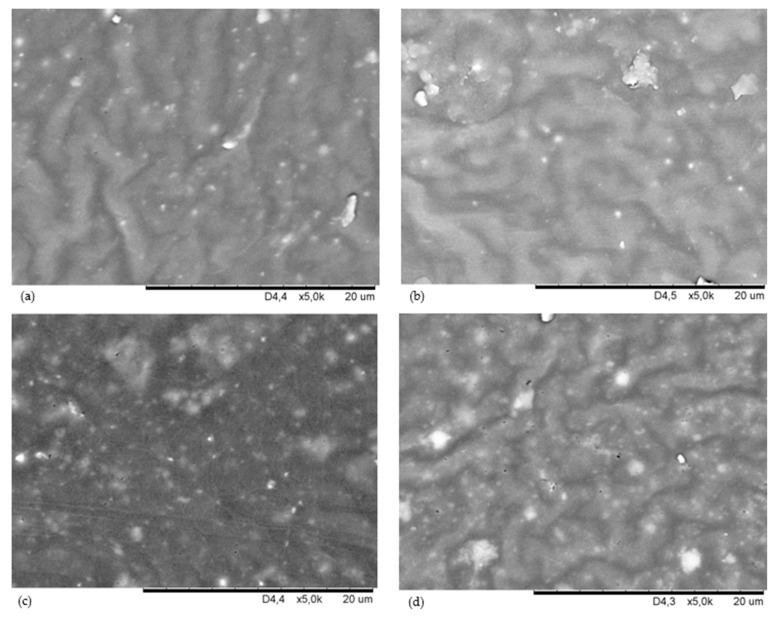
SEM images of the vulcanizate surfaces of: CR/BR/nZn (**a**); CR/BR/nZn/aerosil (**b**); CR/BR/nZn/chalcedonite (**c**); and CR/BR/nZn/sillitin (**d**).

**Figure 2:**
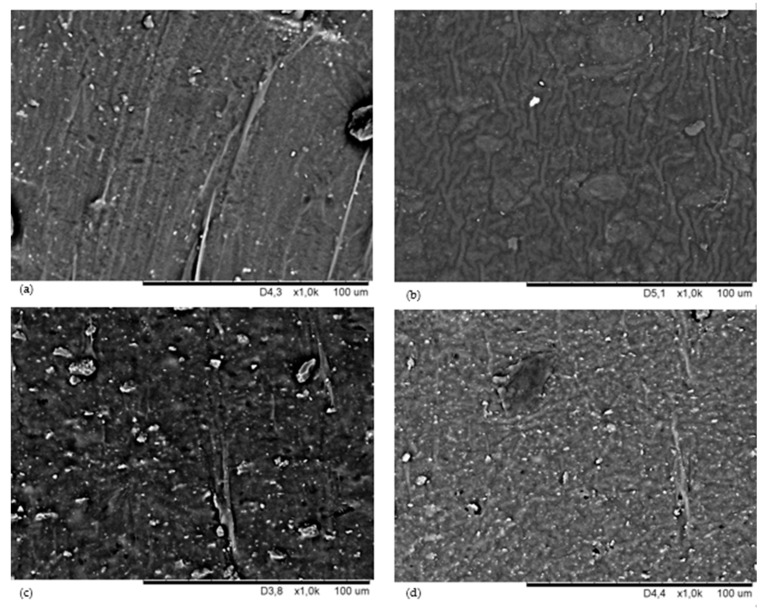
SEM images of the vulcanizate cross-sections of: CR/BR/nZn (**a**); CR/BR/nZn/aerosil (**b**); CR/BR/nZn/chalcedonite (**c**); and CR/BR/nZn/sillitin (**d**).

The most important problem hindering the proper preparation of elastomeric blends is thermodynamic immiscibility of rubbers, which results in heterogeneous morphology of such compositions and, consequently, unsatisfactory properties of the final products. Therefore, it is extremely important to improve the compatibility of components, as well as the course of the vulcanization processes of elastomers [47]. The search for new methods of controlled interelastomer reactions through the appropriate selection of a cross-linking agent capable of a selective reaction with functional groups present in elastomer macromolecules is the most important factor for the effective production of an elastomeric blend. In this research, the use of nano-zinc for the vulcanization of polychloroprene and polybutadiene blends made it possible to connect the chains of both elastomers due to the formation of interelastomer cross-bonds, which determined the final properties of the vulcanizates and affected their surface morphology. The SEM analysis allowed us to evaluate the dispersion of nano-zinc and the fillers used in the CR/BR matrix. SEM images were produced for the samples, which were prepared in two ways: the surfaces of unfilled and filled vulcanizates (Figure 1) and their cross-sections (Figure 2). This was to verify the distribution of individual ingredients in the tested composites in three spatial dimensions. The surface morphologies of the tested vulcanizates at 5 k magnification are presented in Figure 1. The SEM image of the nano-zinc cured CR/BR composite confirmed the proper dispersion of nano-zinc in the rubber phase with its few concentrations (Figure 1a). The SEM image of the CR/BR/nZn vulcanizates filled with aerosil indicates the presence of aggregates of this substance, mostly at the top of the image (Figure 1b). The presence of aerosil deepened the surface roughness with numerous grooves. A similar morphology can be seen in the sillitin-filled vulcanizate, but here, aggregates of this filler were visible throughout the elastomeric mass (Figure 1d). The SEM image of the CR/BR/nZn/Chal vulcanizate showed the lack of a clear boundary between the phases of the elastomers used with marked aggregates of chalcedonite. (Figure 1c). Both chalcedonite and sillitin have similar specific surface areas (10 m^2^ for chalcedonite and 12 m^2^/g for sillitin); however, the SEM images were completely different for compounds containing these fillers, which may indicate different interactions between elastomers (mainly polychloroprene) and these fillers. The dispersion of fillers is determined mainly by the forces of interactions between aggregates and agglomerates, which result from their surface energy. Other factors affecting the dispersion of the filler used are its morphological properties. The compatibility of components of elastomeric blends has a significant impact on the mechanical properties of the final materials.

In Figure 2, the cross-sectional morphologies of the CR/BR blends cross-linked with nano-zinc are presented. The SEM analysis of the cross-section of unfilled sample indicated significant unevenness of the tested samples, especially in the case of the CR/BR/nZn vulcanizate filled with aerosil (Figure 2a,b). Figure 2c,d indicated the presence of chalcedonite and sillitin aggregates (respectively), with larger chalcedonite clusters.

**Figure 3:**
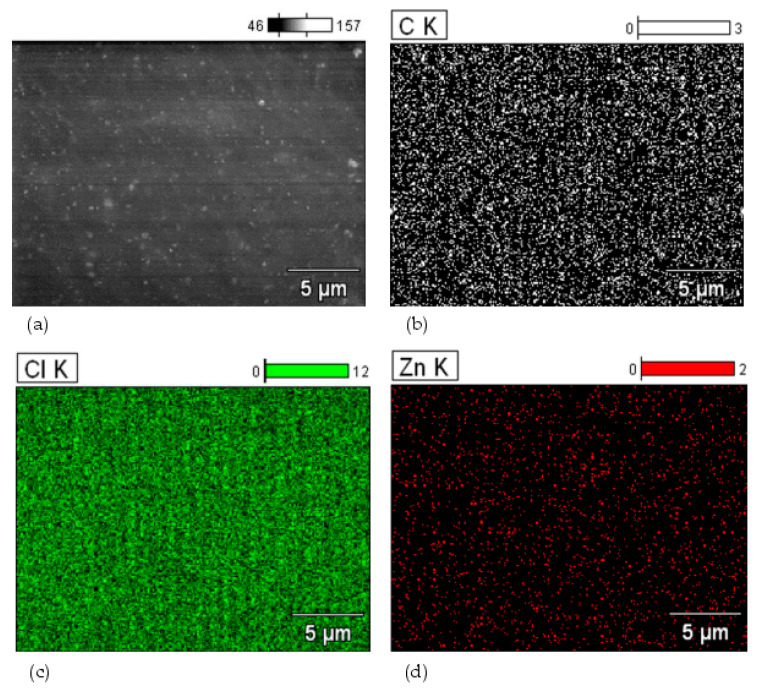
The distribution of elements in selected micro-areas of the CR/BR/nZn vulcanizate’s surface morphology (**a**); distribution of carbon atoms (**b**); distribution of chlorine atoms (**c**); distribution of zinc atoms (**d**); K indicates the electron K shell.

For the unfilled vulcanizate, an additional study using the SEM-EDS method was carried out. Figure 3 shows the distribution of elements in selected micro-areas. The analysis which we performed confirmed the proper dispersion of the indicated elements, including carbon atoms (Figure 3b), chlorine atoms (Figure 3c), and zinc atoms (Figure 3d). Slightly brighter areas, visible in Figure 3d, indicated the presence of small aggregates of nano-sized zinc in the tested CR/BR composites, which was also confirmed by the SEM image produced for this material (Figure 3a).

### 3.3. Dynamic and Mechanical Properties of Unfilled and Filled CR/BR/nZn Vulcanizates

The relationship between the vulcanization characteristics, morphology, and properties typical for cured elastomers as engineering and constructional materials is one of the most important tasks in rubber research. It has been found that the mechanical, dynamic, and thermal properties of vulcanizates are dependent on the amount and distribution of cross-links influenced by the type and concentration of the curing system, cross-links structure, and vulcanization temperature [48]. The curing of elastomers results in distinct growth of tensile strength, tear strength, and hardness. These mentioned ultimate properties occur at different network densities and depend on the type of cross-links [43,49]. Such changes in tensile strength can be associated with changes in the orientation degree of macromolecules during the stretching of vulcanizates. The cross-links created in the initial stage of the vulcanization result in an increase in the strength due to a reduction in plastic transfers of macromolecules and an increase in their orientation in the direction of deforming forces. It should be noted that the changes in the strength during the vulcanization also depend on the type of elastomer that is used. It is worth noting that the structure of an elastomeric product has a great influence on tensile strength, thermal transition, and long-term dynamic fatigue behavior [50]. Similar dependence has been observed in the case of filled vulcanizates. The presence of a filler in the elastomeric compound is very important due to interactions between the filler and the rubber. In these studies, the effect of nano-zinc and selected fillers is evaluated based on tensile properties, hardness, tear resistance, hysteresis losses, Mullins effect, storage and loss modules, and Payne effect. The test results are presented in Table 3.

The produced CR/BR materials cured in the presence of nano-zinc and filled with aerosil, chalcedonite, or sillitin were determined by a wide range of various properties. Unfilled vulcanizate was characterized by tensile strength of 4.99 MPa and a stress value at 100% strain of 0.52 MPa. It is worth emphasizing that the use of nano-sized zinc as a cross-linking agent for CR/BR blends led to vulcanizates with better mechanical properties compared to those of the CR/BR blends (80/20 by wt.) cured in a standard way (i.e., zinc and magnesium oxides), for which TS_b_ = 4.5 MPa [51]. However, it is worth noting that the tensile strength of the CR/BR blends (80/20 by wt.) cross-linked with micro-sized zinc was higher and amounted to 8.56 MPa [32].

The tested CR/BR/nZn composition filled with chalcedonite had insignificantly weaker properties (TS_b_ = 4.77 MPa, S_e100_ = 0.76 MPa). The presence of sillitin slightly improved the tensile strength to 5.08 MPa. The highest TS_b_ value (7.01 MPa) was obtained when aerosil was used. At the same time, the use of aerosil significantly reduced the elongation at break to 390%, while the E_b_ value for unfilled vulcanizate exceeded 1200%; for samples filled with chalcedonite and sillitin, this parameter was 804% and 890%, respectively. Such a clear deterioration of the E_b_ parameter observed for the sample with aerosil indicated lower elasticity, which was also confirmed by the stiffness (S_e100_ = 3.26 MPa) and hardness (52 ^o^Sh A). For the other compositions, the hardness did not exceed 30 ^o^Sh A (Table 3). Such a high hardness of the samples containing silica is caused by the highest degree of cross-linking and parallel morphology creating interpenetrating phases (Figure 1b). The obtained results show that the specific surface area of the filler and the particle size are the factors of decisive importance for improving the mechanical properties of cured rubber products. In this paper, the most active filler was found to be aerosil, which is a nano-sized substance with a large specific surface area. An additional factor increasing the mechanical properties of vulcanizates is the force of interactions between the filler and rubbers. For aerosil with numerous hydroxyl and silanol groups on the surface, these interactions are much stronger than in the case of the two other fillers which were utilized.

All observed mechanical properties can be explained based on the degree of cross-linking (Table 2). An increase in the degree of cross-linking caused by a decrease in the chain mobility under the applied load or stress to which the vulcanizate is subjected led to better mechanical properties. This observation was also in agreement with the tear resistance (T_s_) results of the CR/BR/nZn products. The tear strength is dependent on the network density; thus, for the unfilled sample, the T_s_ value was only 2.91 N/mm. The highest tear resistance (10.45 N/mm) was observed in the case of the CR/Br/nZ/Aer vulcanizate. A surprisingly large T_s_ value (above 8 MPa) was determined for the sillitin-filled product. Again, the weakest and least resistant to tearing (T_s_ = 5.84 N/mm) was the vulcanizate containing chalcedonite. The reason for the poor mechanical properties of the compositions filled with chalcedonite was probably its limited dispersion in the CR/BR matrix and the large size of its particles, which was confirmed by the SEM image (Figure 1c).

Hysteresis is a measure of the amount of mechanical energy that is converted to thermal energy when the sample is deformed. The greater the amount of mechanical energy lost to thermal energy, the more the sample deforms, and it does not reach its original shape even after removal of the external stresses. On the other hand, the calculated Mullins effect is associated with a reduction in stresses during the subsequent identical deformations of filled vulcanizates. This phenomenon occurs when the elastomer–filler interactions are formed or the agglomerate structures of the filler are destroyed. For the CR/BR composite cured with nano-zinc, the lowest hysteresis loss (∆W_1_ = 57.47 N·mm), as a difference of the work between load and unload of the sample during the first cycle, was observed (Table 3). Slightly higher hysteresis losses (62.90 and 74.90 N·mm) were achieved for the samples containing chalcedonite or sillitin. It is certain that the highest ∆W_1_ (449.39 N·mm) value was obtained for the aerosil-filled CR/BR/nZn compounds. Probably, such high hysteresis losses and significant Mullins effect (E_M_ = 79.51%) investigated for the CR/BR/nZn/Aer vulcanizate were caused by the presence of significant interactions of this filler with the CR/BR matrix. The lowest Mullins effect (31.69%) was found for the sample containing chalcedonite, which is consistent with the previously reported poor properties of compositions with this filler.

**Figure 4:**
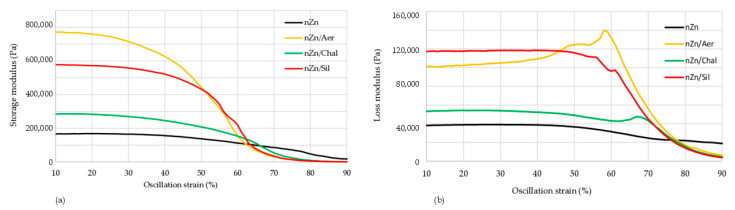
Storage (**a**) and loss (**b**) modules of the unfilled and filled CR/BR/nZn vulcanizates.

The storage modulus (G’) of the rubber products is a measure of their stiffness. Table 3 and Figure 4a present the storage moduli of the unfilled and filled CR/BR/nZn vulcanizates. The addition of aerosil to the tested composites resulted in a clearly significant increase in the storage modulus. All filled vulcanizates had higher G’ values (in case of aerosil used: G’ = 776,209 Pa) compared to the unfilled samples (G’ = 168,377 Pa). Compared to samples containing chalcedonite or sillitin, the G’ parameter of the CR/BR/nZn/Aer compound increased by 170%, and 35%, respectively. The differences between the behavior of the fillers in the elastomer matrix during dynamic measurements can be explained by their different surface properties. The stress–strain relationship in dynamic measurements is related to the type and strength of the filler–elastomer and filler–filler interactions. The filler–rubber interactions are related to the compatibility of the filler with the rubber and are the result of rubber occlusion. This causes the formation of “bonded rubber”, which is trapped inside the aggregates of the filler. Then, the rubber becomes an element of the filler network and increases its effective volume. The filler–filler interactions are the result of the mutual attraction of the filler particles to each other. These interactions testify to the filler’s ability to create its own network (extra-network) in the elastomer matrix and play the most important role in the reinforcement mechanism, especially at a high degree of filling. These forces depend on chemical and physical interactions between filler particle surfaces, network morphology, and filler volume. In the case of aerosil, interactions with elastomers are much stronger than for other fillers. In addition, silica particles show a stronger tendency to interact and a greater tendency to agglomerate, which results from the high value of the specific interaction parameter. This observation was also confirmed by the SEM analysis (Figure 1b and Figure 2b) and hysteresis results (Table 3). A significant G’ value was also observed in the case of vulcanizates containing sillitin, which should also be combined with the cluster formation of this filler in the elastomer matrix visible in the SEM image (Figure 1d and Figure 2d).

Changes in the loss modulus (G”) versus the oscillation strain are shown in Figure 4b. The loss modulus, and, thus, the amount of energy dissipated during the dynamic deformation of the sample, depends on two processes: destruction and reconstruction of the filler structure. The more of the filler structure is destroyed and rebuilt during one deformation cycle, the greater the loss modulus becomes. The more active the filler, the greater the *G*”_max_ modulus, which is a function of the filler’s interface between phases. Therefore, increasing the distance between the filler agglomerates and/or aggregates or the binding of the filler particles to the rubber reduces the *G*”_max_ value.

The Payne effect (ΔG’) is associated with a noticeable decrease in the storage modulus and a peak of the loss modulus, along with an increase in the strain amplitude, in dynamic tests of elastomeric composites, as well as with the destruction of the filler network in the elastomer matrix, which is a key factor reinforcing the produced rubber material. The Payne effect depends on the destruction of the secondary structure of the filler during dynamic measurements of filled vulcanizates. The distribution of the filler particles in the elastomer matrix is also an important factor. Composites, in which the particles are well dispersed, are characterized by a weak Payne effect and a small effect of temperature on the storage modulus (G’), in contrast to the samples containing large clusters of particles in the form of aggregates and agglomerates. As expected, the Payne effect was the smallest (150,425 Pa) for the vulcanizate without any filler, while the use of aerosil led to a ΔG’ of 775,709 Pa. These results confirm that the greatest interactions arise between the CR/BR matrix and aerosil, resulting in good mechanical properties and a high degree of cross-linking (Table 3). In the case of the sample with chalcedonite, it is likely that there were limited interactions of this filler with elastomers, translating into worse dynamic properties.

**Figure 5:**
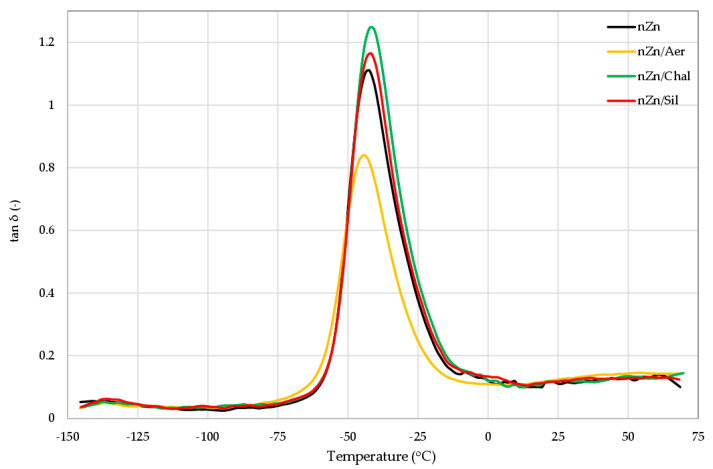
Loss factor (tan δ) curves versus temperature of the unfilled and filled CR/BR/nZn vulcanizates.

Dynamic mechanical analysis (DMA) was used to measure the viscoelastic properties of the tested composites as a function of temperature. This method allowed us to evaluate the effects of the utilized fillers on the glass transition temperature of the CR/BR blends cross-linked with nano-zinc and their viscoelastic properties. The DMA spectra are shown in Figure 5, and the results are presented in Table 3. On the DMA spectra, only the glass transitions of the tested vulcanizates were observed; they were recorded as the maximum peaks of the mechanical loss coefficient (tan δ). The glass transition temperature (T_g_) is the temperature at which the maximum of tan δ occurred on the DMA curve. The T_g_ value determined for the CR/BR composites cross-linked with nano-zinc was −42.25 °C. Very similar T_g_ values were observed for vulcanizates filled with chalcedonite (T_g_ = −42.25 °C) or sillitin (T_g_ = −41.65 °C). The CR/BR/nZn/Aer vulcanizate showed the lowest glass transition temperature (T_g_ = −44.25 °C). The sample containing chalcedonite was characterized by the highest value of tan δ (1.25). This results from the greater flexibility of this vulcanizate. The presence of aerosil in the tested compositions significantly reduced the height of the tan δ peak (0.84). This result indicates that the aerosil-filled vulcanizate achieved the highest stiffness, which is consistent with the previously described results, e.g., stress at 100% strain, elongation at break, and hardness (Table 3).

### 3.4. Determination of Thermal Properties of Unfilled and Filled CR/BR/nZn Vulcanizates

**Figure 6:**
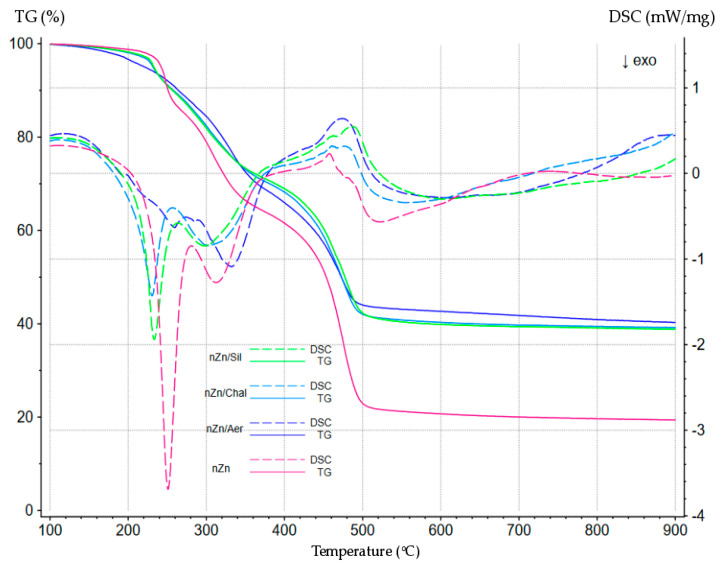
The TG and DSC curves of the unfilled and filled CR/BR blends cross-linked with nano-sized zinc (nZn).

The thermal properties of the CR/BR/nZn products were studied by means of simultaneous thermal analysis (STA), which generally refers to the simultaneous application of thermogravimetry (TG) and differential scanning calorimetry (DSC) to one and the same sample in a single instrument. The CR/BR/nZn vulcanizates were subjected to pyrolysis, i.e., degradation occurring at high temperatures in an oxygen-free atmosphere. The mass changes (TG) and thermal effects (DSC) occurring during the heating from 50 °C to 1000 °C are shown in Figure 6.

The TG curve shows that pyrolysis and decomposition of the tested materials began at a temperature of approx. 160 °C. Several stages of this decomposition were visible. It is interesting that unfilled CR/BR/nZn vulcanizate decomposed later, i.e., it was the most thermally stable and began to rapidly lose weight only at 250 °C. The presence of fillers, regardless of their structure, caused vulcanizates to decompose more quickly. This is most likely due to the presence of water chemically bound to the tested fillers. Water molecules detach at elevated temperatures, so the mass of the sample decreases, and additionally, the released water initiates chemical degradation processes.

In the first period of pyrolysis of the unfilled and filled CR/BR/nZn composites (200–260 °C), exothermic effects were observed on the DSC curves, which were indicated by the downward peaks that shifted relative to each other. Their analysis confirmed that the unfilled sample was more thermally stable than the vulcanizates filled with chalcedonite or sillitine. A different DSC curve was observed for the sample filled with aerosil. Surprisingly, in this range, there were no visible endothermic effects, which should be recorded on the TG curve during the decomposition of the sample.

**Figure 7:**
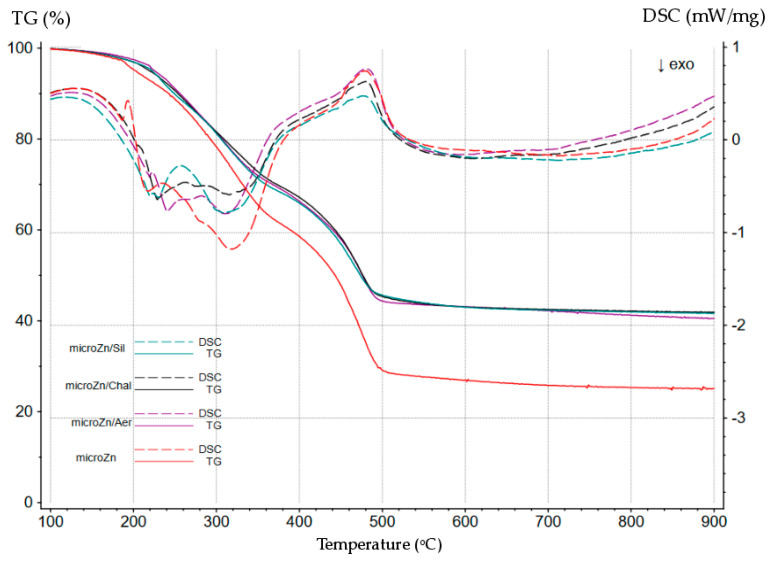
The TG and DSC curves of the unfilled and filled CR/BR blends cross-linked with micro-sized zinc (Zn).

The results of the STA analysis were so astonishing that it was decided that we would perform such tests for the CR/BR compositions cross-linked with micro-sized zinc (Zn) as well (their properties have been described in an earlier publication [32]). The course of TG and DSC for the CR/BR/Zn vulcanizates (80/20/2.5 by wt.) is shown in Figure 7. It is clearly visible that the TG and DSC curves for the compositions cross-linked with micro-sized zinc (CR/BR/Zn) were different than for the blends cured with nano-sized zinc (CR/BR/nZn). First of all, the TG curves were more reproducible for the CR/BR/Zn vulcanizates. The DSC curves of the CR/BR/Zn samples were also dominated by exothermic effects (in the temperature range of 200–400 °C), but some of these became minor endothermic effects (430–500 °C). The most surprising fact was that the exothermic effects occured in two areas (the first in the temperature range of 200–400 °C, the second in the temperature range of 500–800 °C). However, regardless of the size of the zinc particles used to cross-link the CR/BR blends, it was found that the fillers (chalcedonite, sillitin, and aerosil) slowed down the reactivity of both nano-zinc and micro-zinc.

**Figure 8:**
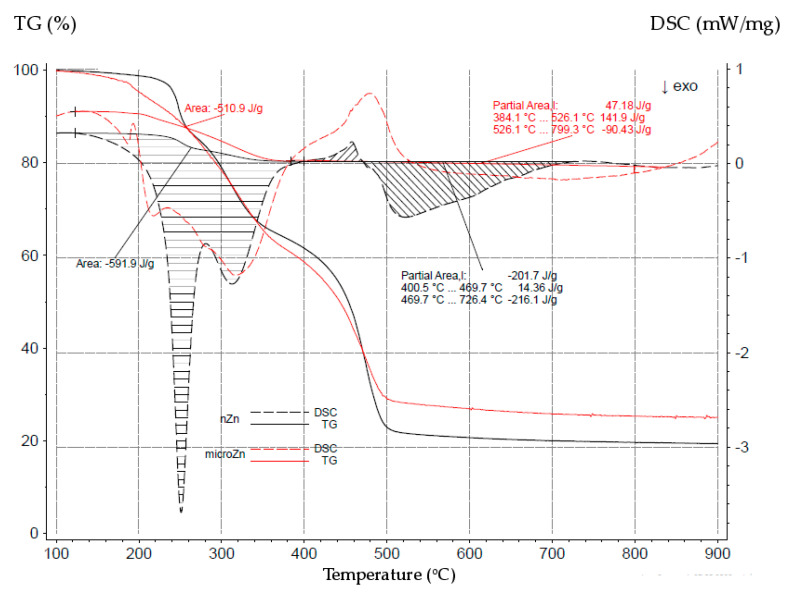
The TG and DSC curves of the unfilled CR/BR composites cross-linked with nano-sized zinc (nZn) or micro-sized zinc (Zn).

In addition, unfilled compositions cross-linked with nano-sized zinc or micro-sized zinc were compared in order to better understand the phenomena occurring during the pyrolysis of the CR/BR/nZn and CR/BR/Zn vulcanizates (Figure 8). Significant differences in the courses of the TG and DSC curves are visible. In Figure 8, numerical values are added to better demonstrate these differences. The first area was similar. The determined enthalpy for the first exothermic effect was ΔH = −591.9 J/g (for the CR/BR/nZn sample) and ΔH = −510.9 J/g (for the CR/BR/Zn sample). For the vulcanizate containing micro-sized zinc, the dynamics of heat release decreased, but it was a similar process, indicating that a similar reagent participated in these reactions. However, it should be emphasized that the process with nano-sized zinc was faster than that with micro-sized zinc. These observations clearly indicated a chemical reaction involving nano-zinc or micro-zinc. It is obvious that at low temperatures (approx. 250 °C)—in the case of the CR/BR compositions cross-linked with nano-zinc—there are many smaller particles that are in contact with the potential reactant (most likely chlorine atoms), which determines the high rate of such a reaction. On the other hand, micro-zinc must react inward, so the process is slower. Hence, the difference in the DSC curves in the range of 200–380 °C were visible. In the further temperature range, the endothermic effect was shown (greater for the CR/BR/Zn vulcanizates), because the largest loss of mass associated with the degradation of the studied composites was observed (TG curves).

At the first mass loss (250–320 °C), the endothermic effect should be visible, but it was most likely hidden because the exothermic effect superimposes it. Therefore, the DSC curve shows rather a thermal balance, since the various chemical processes coincide with each other. The absence of an exothermic effect shown in the temperature range of 350–500 °C does not mean that this effect did not occur there, because the reaction of zinc with chlorine was still taking place. In this area, the exothermic effect was covered by a greater endothermic effect, because the largest losses of the sample were visible here. In addition, the TG curve shows that the tested samples were completely decomposed (residue of approx. 20–30% by weight) at a temperature of approx. 500 °C. On the other hand, the DSC curve shows an exothermic effect in this area, indicating a chemical reaction taking place in the sample remaining after the disintegration. Moreover, the exothermic effects of the CR/BR/nZn and CR/BR/Zn samples differed significantly. For the sample with micro-sized zinc, the exothermic effect was more extended in time and required a higher temperature (up to 700 °C), and the exothermic effect for the composition containing nano-sized zinc was faster and occurred at a lower temperature (approx. 520 °C).

### 3.5. Flammability and Toxicity of Unfilled and Filled CR/BR/nZn Vulcanizates

The combustion of elastomeric materials often leads to the generation of liquid products of their thermal decomposition, increasing contact with oxygen, and an additional source of heat. A characteristic feature of the tested elastomers (especially polychloroprene) is their susceptibility to thermo-cross-linking, which can limit the formation of liquid products during their combustion. The vulcanization of the CR/BR compositions in the presence of nano-sized zinc made the oxygen index (OI) comparable with the parameters determined in such compounds in our previous works [23,24,25,26,28,29,30,31,32], and only slightly higher than the OI value for similar blends cured with micro-sized zinc [32]. The obtained results indicated that the use of nano-zinc as a cross-linking substance for the CR/BR composites led to the formation of flame-retardant products. Such a high OI value in the investigated samples was due to interelastomer reactions occurring during the unconventional vulcanization of the CR/BR blends in the presence of nano-sized zinc. In addition, as expected, the use of fillers additionally increased the oxygen index to a value exceeding 37.5% (Table 4).

**Figure 9:**
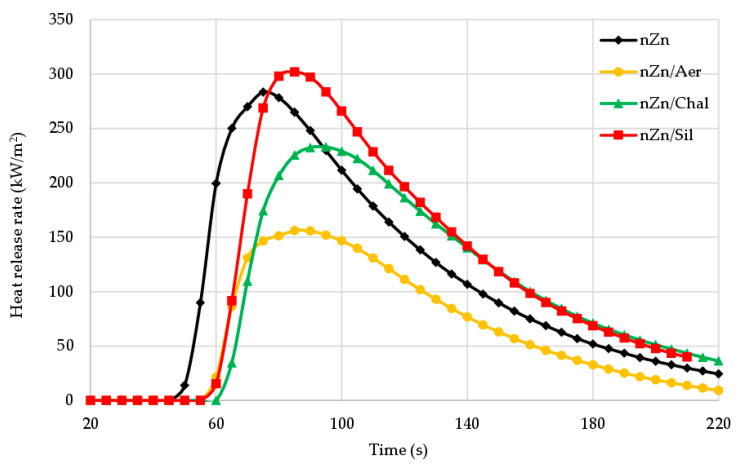
Heat release rate (HRR) curves of the unfilled and filled CR/BR/nZn vulcanizates.

Cone calorimetry studies were conducted to perform a more detailed assessment of the flammability of the manufactured products. The HRR_max_ parameter (i.e., heat release rate) defines the maximum rate of heat released during the combustion of rubber products. Based on this parameter, it can be concluded that the vulcanization of the CR/BR compositions in the presence of nano-zinc led to materials characterized by low fire hazard. The course of changes in the HRR parameter is shown in Figure 9. The maximum heat release rate of the unfilled vulcanizates was only 283 kW/m^2^. For the aerosil-filled CR/BR/nZn, the HRR_max_ value decreased to 156 kW/m^2^. The presence of chalcedonite or sillitin in the vulcanizates led to HRR_max_ values equal to 233 kW/m^2^ or 302 kW/m^2^, respectively (Table 4). As shown in Figure 9, from the heat release rate curves, it is indicated that the HRR value increased rapidly for the sample without a filler or for the sample containing sillitin. It is worth highlighting that the heat release rate during the combustion of all tested materials was visibly lower compared to other elastomeric goods. For comparison, standard vulcanized ethylene–propylene–diene rubber (EPDM), both unfilled and filled with basalt fibers, is characterized by HRR values equal to 1819 kW/m^2^ and 648 kW/m^2^, respectively [1].

**Figure 10:**
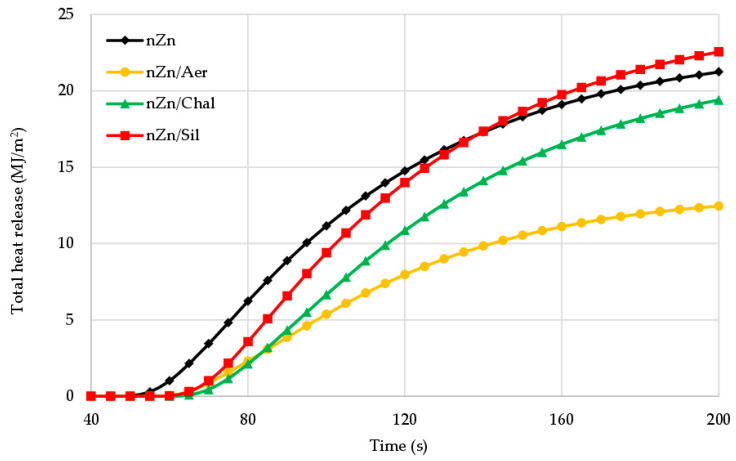
Total heat release (THR) curves of the unfilled and filled CR/BR/nZn vulcanizates.

The THR parameter, i.e., total heat release, is another important factor to be analyzed when the flammability of materials is discussed. The THR value for the CR/BR composite cured with nano-was was only 19.8 MJ/m^2^ (Table 4). The presence of tested fillers in the CR/BR/nZn vulcanizates clearly reduced the total heat release to 10.5 MJ/m^2^, 16.0 MJ/m^2^, and 18.0 MJ/m^2^, respectively, for aerosil, chalcedonite and sillitin. Figure 10 confirms that the aerosil-filled compound exhibited a much lower total heat release than other vulcanizates. For comparison, the THR values for butadiene–acrylonitrile rubber, styrene–butadiene rubber, and butadiene rubber are equal to 98.71 MJ/m^2^, 87.88 MJ/m^2^, and 84.5 MJ/m^2^, respectively [9].

**Figure 11:**
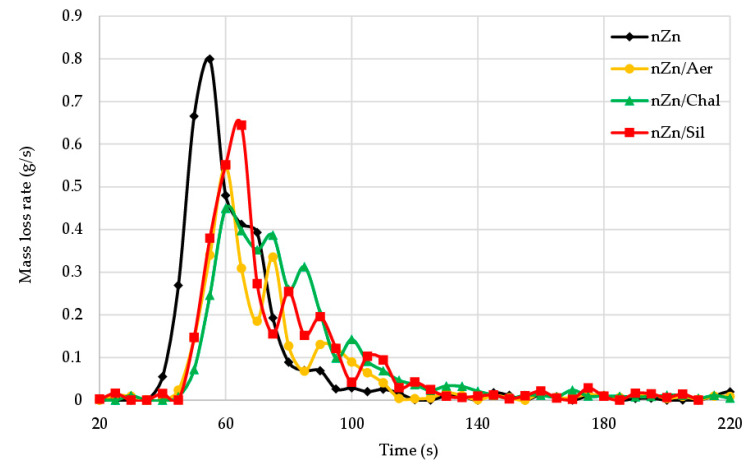
Mass loss rate (MLR) curves of the unfilled and filled CR/BR/nZn vulcanizates.

The average mass loss rate (AMLR), which proves the dynamics of material combustion, reached the highest value (55.38 g/m^2^·s) in the case of the CR/BR/nZn vulcanizate, but the presence of aerosil, chalcedonite, or sillitin resulted in a reduction of AMLR to 25.94 g/m^2^·s, 32.88 g/m^2^·s, or 33.54 g/m^2^·s, respectively. The mass loss rate (MLR) versus the incineration time of the unfilled and filled tested samples is shown in Figure 11. It is clearly visible that the mass loss rate was much smaller for the filled samples, which indicates the positive effect of all the fillers used. In addition, the mass loss rate depended on the fillers’ presence in the rubbers and their types (Table 4).

At a sufficiently high temperature, intensive cracking of chemical bonds in the macromolecules of burnt vulcanizates takes place. Most of the bonds are broken, leading to the destruction of the polymer. The destruction mechanism depends on the chemical structure of the macromolecules, the rate of heating, and the thermal effects of the reaction. The value of the dissociation energy of chemical bonds has a significant impact on the thermal stability of the polymer and its resistance to fire. In the CR/BR/nZn vulcanizates, there are chemical bonds with higher dissociation energy than, for example, in composites cross-linked with organic peroxide or sulfur, which translates into their significant resistance to fire. The results obtained from cone calorimetry determine the fire hazard related to the fire propagation rate (1/t_flashover_) [52]. This is described as the inverse time to reach the flashover effect (Formula (8)). The cross-linking of the CR/BR blends with nano-zinc provided the products with a low fire hazard (1/t_flashover_ = 7.27 kW/m^2^·s). The fire propagation rate for composites containing sillitin was slightly lower (t_flashover_ = 6.43 kW/m^2^·s), but in the case of vulcanizates filled with aerosil or chalcedonite, this parameter was lower by 53% or 40%, respectively (Table 4). It is worth noting that the 1/t_flashover_ values of all CR/BR/nZn vulcanizates were slightly higher as this index was determined for the zinc-cured CR/BR blends, whose corresponding values amounted to 4.67 kW/m^2^·s [32]. However, the tested nano-zinc-cured CR/BR blends posed a significantly lower fire hazard than butadiene rubber or butadiene–acrylonitrile rubber (1/t_flashover_ equal to 56.37 and 31.68 kW/m^2^·s, respectively) [9]. Thus, the obtained results confirmed that all of the nano-zinc-cured CR/BR products were non-flammable and posed a low fire hazard.

The FIGRA parameter shows the ratio of the maximum heat release rate to the time to maximum heat release rate. The FIGRA index was similar (approx. 3.67 kW/m^2^·s) for the sample without a filler and for the sample containing sillitin, whereas the CR/BR/nZn vulcanizates filled with chalcedonite and aerosil were characterized by a FIGRA of 2.45 kW/m^2^·s or 1.84 kW/m^2^·s. The maximum average heat release rate (MARHE) of the sample filled with aerosil was only 70.3 kW/m^2^, whereas this index for the CR/BR blends cured with nZn was 124.1 kW/m^2^·s (the same value was observed in the case of sillitin). The analysis of the obtained results confirmed that the cross-linking of the polychloroprene and polybutadiene compositions with nano-zinc led to the production of materials with low fire risk. In addition, the use of aerosil had the greatest impact on further reducing the fire hazard. Most likely, during combustion, aerosil creates a thermally stable boundary layer on the vulcanizate surface, which significantly hinders the flow of mass and energy between the rubber product and the flame.

**Figure 12:**
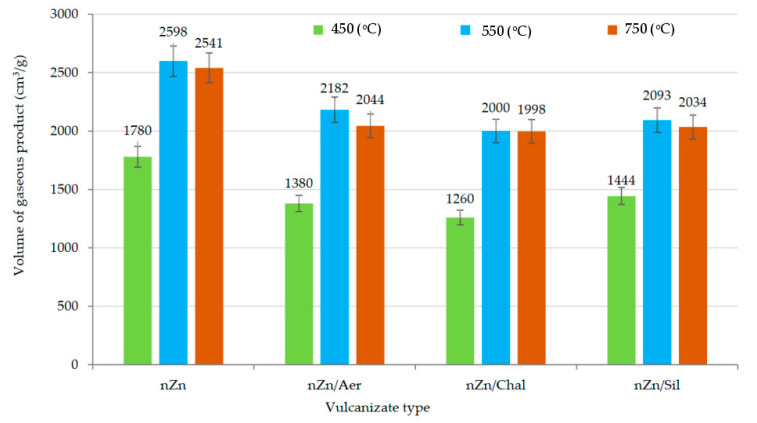
The volume of gaseous products formed during the combustion and decomposition of the unfilled and filled CR/BR/nZn vulcanizates at temperatures of 450, 550, and 750 °C.

The toxicity of gaseous substances generated as a result of the decomposition and combustion of the CR/BR/nZn products was also an object of this research (Table 5). The emissions of carbon dioxide (CO_2_), carbon monoxide (CO), nitrogen (IV) oxide (NO_2_), sulfur (IV) oxide (SO_2_), hydrogen chloride (HCl), and hydrogen cyanide (HCN) were calculated. The toxicometric index (W_LC50SM_, Formula (11)) is a basic parameter, because it includes the concentrations of all six gases emitted at temperatures of 450 °C, 550 °C, and 750 °C [9]. It follows that the highest emission values were registered for carbon oxides, especially for CO_2_, regardless of the decomposition temperature and the sample type. The carbon dioxide emission for the filled samples was slightly lower (approx. 1.23 g/g for 450 °C, approx. 1.79 g/g for 550 °C, and approx. 2.35 g/g for 750 °C) than for the unfilled compound (1.70 g/g for 450 °C, 2.36 g/g for 550 °C, and 2.91 g/g for 750 °C), but the filler type did not significantly affect the amount of gas emissions. The value of the toxicimetric index was influenced by the emission of SO_2_ (0.01 g/g for all samples) and HCl (the highest emission for an unfilled sample: 0.17 g/g for 450 °C, 0.19 g/g for 550 °C, and 0.19 g/g for 750 °C). A potential source of HCl is chlorine bound to the macromolecules of the polychloroprene. Our observations were also confirmed by the volumes of gaseous products (Figure 12), which were higher for the CR/BR/nZn product and lowest for the vulcanizate filled with chalcedonite. Unfortunately, the WLC_50SM_ parameter indicates that the gaseous destructs formed during the decomposition processes of tested composites belong to the category of very toxic materials (Table 6) [32]. The smaller the WLC_50SM_ parameter, the greater the toxicity of the material. From this point of view, the incorporation of the tested fillers into CR/BR/nZn compositions is beneficial for the environment.

## 4. Conclusions

In summary, polychloroprene and polybutadiene (CR/BR) composites can be effectively cross-linked with nano-sized zinc (nZn). Under the influence of the vulcanization of CR/BR blends in the presence of nano-zinc, both elastomers had good compatibility. This makes it possible to interbond rubber chains due to the generation of interelastomer cross-links. The filler type had a large impact on the degree of cross-linking of the CR/BR/nZn, the morphology, the mechanical–dynamic properties, and the fire resistance (HHR_max_ = 156.15 kW/m^2^) of the final products. Among the fillers which we used, the presence of aerosil ensured the highest degree of cross-linking (α_c_ = 0.16, Q_v_ = 6.21 mL/mL, 2C_1_ = 3.22 kG/cm^2^), the best mechanical properties (TS_b_ = 7.01 MPa, HA = 52 ^o^ShA, T_s_ = 10.45 N/mm), and the highest Payne effect (ΔG’ = 775,709 Pa) and Mullins effect (E_M_ = 79.51%). It confirms the superior interactions between this filler and elastomer matrix. Our analysis of the thermal properties of CR/BR/nZn vulcanizates by the STA method demonstrated that unfilled samples had better thermal stability compared to filled compositions. However, the use of nano-sized zinc had a significant impact on the course of TG and DSC curves. This result was obtained by performing a comparative STA analysis for CR/BR blends cross-linked with nano-sized zinc (nZn) or micro-sized zinc (Zn). The flammability study indicates that all produced vulcanizates were characterized by a high oxygen index (OI > 29%), which allows them to be classified as non-flammable materials. The most important advantage of the manufactured materials was their low fire hazard (1/t_flashover_ = 3.47–7.27 kW/m^2^·s). The production of CR/BR/nZn products, both unfilled and filled, with limited susceptibility to burning is positive for safety reasons for humans, as well as for ecological reasons. Therefore, it is in line with the Sustainable Development Goals (The 2030 Agenda for Sustainable Development).

## Figures and Tables

**Table 1:** Compositions of the CR/BR/nZn blends.

Ingredient	Ingredient Amount (phr)			
CR	80	80	80	80
BR	20	20	20	20
SA	1	1	1	1
Aer	-	30	-	-
Chal	-	-	30	-
Sil	-	-	-	30
nZn	2.5	2.5	2.5	2.5

CR—polychloroprene, BR—polybutadiene, SA—stearic acid, Aer—aerosil, Chal—chalcedonite, Sil—sillitin, nZn—nano-zinc, phr—parts per hundred of rubber.

**Table 2:** Cross-linking parameters of the unfilled or filled CR/BR/nZn blends.

Parameter	Symbol			
	nZn	nZn/Aer	nZn/Chal	nZn/Sil
t_02_ (min)	1.98	2.42	3.29	5.38
t_90_ (min)	26.31	26.83	30.63	27.60
T_min_ (dN·m)	0.56	4.09	0.58	0.83
∆T_30_ (dN·m)	3.67	7.95	5.07	4.84
CRI (min^−1^)	4.11	4.10	3.66	4.50
Q_v_ (mL/mL)	9.70	6.21	6.90	8.72
α_c_	0.10	0.16	0.14	0.11
2C_1_ (kG/cm^2^)	1.45	3.22	2.28	1.87
2C_2_ (kG/cm^2^)	0.84	1.17	0.90	1.56

t_02_—scorch time; t_90_—cure time; T_min_—minimal torque; ∆T_30_—torque increment after 30 min of heating; CRI—cure rate index; Q_v_—equilibrium swelling degree; α_c_—cross-linking degree; 2C_1_, 2C_2_—first and second Mooney–Rivlin elasticity constant.

**Table 3:** The mechanical–dynamic properties of the unfilled or filled CR/BR/nZn vulcanizates.

Parameter	Symbol			
	nZn	nZn/Aer	nZn/Chal	nZn/Sil
S_e100_ (MPa)	0.52 ± 0.02	3.26 ± 0.24	0.76 ± 0.04	0.94 ± 0.05
S_e200_ (MPa)	0.67 ± 0.03	4.75 ± 0.26	1.16 ± 0.05	1.45 ± 0.06
S_e300_ (MPa)	0.83 ± 0.05	6.07 ± 0.25	1.57 ± 0.05	1.96 ± 0.09
TS_b_ (MPa)	4.99 ± 0.36	7.01 ± 0.09	4.77 ± 0.27	5.08 ± 0.42
E_b_ (%)	1250 ± 2	390 ± 11	804 ± 38	890 ± 53
HA (^o^Sh A)	25 ± 1	52 ± 3	29 ± 2	26 ± 1
T_s_ (N/mm)	2.91 ± 0.15	10.45 ± 0.87	5.84 ± 0.46	8.05 ± 0.38
ΔW_1_ (N·mm)	57.5 ± 5.1	449.4 ± 36.2	62.9 ± 4.5	74.9 ± 6.3
E_M_ (%)	49.5 ± 3.3	79.5 ± 6.9	31.7 ± 3.9	36.3 ± 2.6
G’_max_ (Pa)	168,377	776,209	286,103	576,414
G”_max_ (Pa)	39,071	140,123	54,239	118,483
ΔG’ (Pa)	150,425	775,709	285,226	457,931
T_g_ (°C)	−42.25	−44.25	−42.25	−41.65
tan δ_Tg_ (-)	1.11	0.84	1.25	1.16

S_e100_, S_e200_, S_e300_—stress at 100%, 200%, 300% strain; TS_b_—tensile strength; E_b_—elongation at break; HA—hardness; T_s_—tear resistance; ΔW_1_—hysteresis losses; E_M_—Mullins effect; G’_max_—storage modulus; G”_max_—loss modulus; ΔG’—Payne effect.

**Table 4:** Parameters characterizing the flammability and fire hazard of the unfilled and filled CR/BR/nZn vulcanizates, determined by oxygen index and cone calorimetry.

Parameter	Symbol			
	nZn	nZn/Aer	nZn/Chal	nZn/Sil
OI (%)	29.8 ± 1.5	>37.5	>37.5	>37.5
TTI (s)	39 ± 2	45 ± 2	53 ± 2	47 ± 2
TTF (s)	170 ± 2	154 ± 2	156 ± 2	145 ± 2
HRR_max_ (kW/m^2^)	283 ± 10	156 ± 10	233 ± 10	302 ± 10
tHRR_max_ (s)	75 ± 8	85 ± 8	95 ± 8	85 ± 8
THR (MJ/m^2^)	19.8 ± 2.0	10.5 ± 2.0	16.0 ± 2.0	18.0 ± 2.0
EHC (MJ/kg)	9 ± 0.5	7 ± 0.5	9 ± 0.5	10 ± 0.5
EHC_max_ (MJ/kg)	77 ± 5	69 ± 5	67 ± 5	65 ± 5
MLR_max_ (g/s)	0.799 ± 0.5	0.548 ± 0.5	0.449 ± 0.5	0.644 ± 0.5
AMLR (g/m^2^·s)	55.38 ± 5	25.94 ± 5	32.88 ± 5	33.54 ± 5
1/t_flashover_ (kW/m^2^·s)	7.27 ± 0.5	3.47 ± 0.5	4.39 ± 0.5	6.43 ± 0.5
FIGRA (kW/m^2^·s)	3.78 ± 1.2	1.84 ± 1.2	2.45 ± 1.2	3.55 ± 1.2
MARHE (kW/m^2^)	124.1 ± 10	70.3 ± 10	103.1 ± 10	124.3 ± 10

OI—oxygen index, TTI—time to ignition, TTF—time to flameout, HRR_max_—maximum heat release rate, tHRR_max_—time to maximum heat release rate, THR—total heat release, EHC—effective heat of combustion, EHC_max_—max. effective heat of combustion, MLR_max_—maximum mass loss rate, AMLR—average mass loss rate, 1/t_flashover_—fire propagation rate, FIGRA = HRR_max_/tHRR_max_, MARHE—maximum average heat release rate.

**Table 5:** Specific emission of gaseous products formed during the decomposition and combustion of the unfilled and filled CR/BR/nZn vulcanizates at temperatures of 450, 550, and 750 °C.

Symbol	T (°C)	Emission (g/g)					
		CO_2_	CO	NO_2_	SO_2_	HCl	HCN
nZn	450	1.70 ± 0.16	0.28 ± 0.02	0.00 ± 0.00	0.01 ± 0.00	0.17 ± 0.02	0.00 ± 0.00
	550	2.36 ± 0.17	0.42 ± 0.05	0.00 ± 0.00	0.01 ± 0.00	0.19 ± 0.02	0.00 ± 0.00
	750	2.91 ± 0.40	0.02 ± 0.01	0.00 ± 0.00	0.01 ± 0.00	0.19 ± 0.06	0.00 ± 0.00
nZn/Aer	450	1.26 ± 0.24	0.24 ± 0.04	0.00 ± 0.00	0.01 ± 0.00	0.12 ± 0.02	0.00 ± 0.00
	550	1.94 ± 0.11	0.34 ± 0.02	0.00 ± 0.00	0.01 ± 0.00	0.15 ± 0.01	0.00 ± 0.00
	750	2.34 ± 0.21	0.02 ± 0.01	0.00 ± 0.00	0.01 ± 0.00	0.14 ± 0.01	0.00 ± 0.00
nZn/Chal	450	1.21 ± 0.13	0.23 ± 0.04	0.00 ± 0.00	0.00 ± 0.00	0.11 ± 0.03	0.00 ± 0.00
	550	1.68 ± 0.19	0.41 ± 0.05	0.00 ± 0.00	0.01 ± 0.00	0.14 ± 0.02	0.00 ± 0.00
	750	2.36 ± 0.03	0.00 ± 0.00	0.00 ± 0.00	0.01 ± 0.00	0.14 ± 0.01	0.00 ± 0.00
nZn/Sil	450	1.23 ± 0.05	0.30 ± 0.04	0.00 ± 0.00	0.01 ± 0.00	0.12 ± 0.01	0.00 ± 0.00
	550	1.76 ± 0.20	0.40 ± 0.03	0.00 ± 0.00	0.01 ± 0.00	0.15 ± 0.02	0.00 ± 0.00
	750	2.36 ± 0.25	0.00 ± 0.00	0.00 ± 0.00	0.01 ± 0.00	0.15 ± 0.03	0.00 ± 0.00

**Table 6:** Toxicometric indicators of the unfilled and filled vulcanizates determined at temperatures of 450, 550, and 750 °C.

Symbol	T (°C)	W_LC50_ (g/m^3^)						W_LC50M_	W_LC50SM_
		CO_2_	CO	NO_2_	SO_2_	HCl	HCN		
nZn	450	116 ± 11	14 ± 1	0 ± 0	110 ± 18	6 ± 1	249 ± 22	3.8 ± 0.3	3.90
	550	84 ± 6	9 ± 1	0 ± 0	79 ± 9	5 ± 0	0 ± 0	3.1 ± 0.3	
	750	68 ± 10	351 ± 491	0 ± 0	97 ± 5	6 ± 2	0 ± 0	4.8 ± 1.3	
nZn/Aer	450	157 ± 29	16 ± 3	0 ± 0	124 ± 2	9 ± 1	489 ± 28	5.1 ± 0.8	4.99
	550	101 ± 6	11 ± 1	0 ± 0	92 ± 15	7 ± 1	0 ± 0	3.8 ± 0.2	
	750	84 ± 8	217 ± 121	0 ± 0	118 ± 18	7 ± 0	0 ± 0	6.0 ± 0.3	
nZn/Chal	450	162 ± 17	16 ± 3	0 ± 0	136 ± 3	9 ± 3	402 ± 66	5.3 ± 1.2	5.14
	550	118 ± 14	9 ± 1	0 ± 0	100 ± 11	7 ± 1	0 ± 0	3.8 ± 0.5	
	750	83 ± 1	4395 ± 1442	0 ± 0	128 ± 11	7 ± 0	0 ± 0	6.3 ± 0.3	
nZn/Sil	450	160 ± 7	13 ± 2	0 ± 0	120 ± 9	8 ± 1	301 ± 39	4.6 ± 0.5	4.79
	550	112 ± 13	9 ± 1	0 ± 0	94 ± 17	7 ± 1	0 ± 0	3.7 ± 0.2	
	750	84 ± 7	2129 ± 1502	0 ± 0	113 ± 5	7 ± 1	0 ± 0	6.1 ± 1.1	

W_LC50_—the maximum toxic concentration of gaseous product, W_LC50M_—the resultant of the W_LC50_ index of individual products, W_LC50SM_—the arithmetic average of W_LC50M_ indices.

## Data Availability

Data sharing is not applicable.
